# Health or Fashion?

**Published:** 1902-05-03

**Authors:** 


### Health or Fashion?

There chanced to arrive upon our table, almost at the same moment, the April number of the *Journal of the Sanitary Institute*, containing a very admirable and instructive report by Miss Alice Ravenhill upon the teaching of hygiene in the schools of the United States of America, and a number for the same date of a journal which shall be nameless, but which is devoted to the illustration and description of the latest vagaries in the fashion of female—we will not say feminine—habiliments. It is difficult to imagine a more complete contrast than that which exists between Miss Ravenhill's account of costume as it is guided by knowledge, and the *modiste's* account of it as controlled by that secret tribunal of drapers, milliners, and manufacturers by which, in the last resort, the clothing of great ladies and of their imitators is designed in whatever manner may promise to be most profitable to the respective callings of the several members of the junta. Miss Ravenhill tells us that in many of the higher class American private schools for girls a school dress is insisted upon, and she describes the arrangements at the Girls' Classical High School at Indianapolis as an example. It is believed, she says, "that such a dress discourages extravagance, and prevents the comparisons and discussions of clothes which often distract the attention of school-girls from their proper studies." At the University of Michigan every student is expected to apply her energies to the development of symmetry and grace and of ready physical control, as well as to the improvement of her action and carriage. As one aid to the attainment of these ends, her attention is carefully directed to her reflection in a large mirror, before which is placed a frame divided by string netting into half-inch squares; so that, if one of the string lines be carefully followed across, the slightest deviation from a uniform level of shoulders, for instance, is detected conclusively by the individual concerned. Measurements are employed to determine whether any such deviation arises from a difference in the length of the legs, or from a faulty position of the trunk; and, when this detail is decided, advice is given as to habitual posture, and as to the physiological effects of different attitudes. It would be hardly possible, we think, for a girl thus trained to a recognition of real symmetry and of true grace, to sacrifice either one or the other, as soon as she was emancipated from the restraints of school or college, for the sake of placing her neck beneath the yoke of so-called fashion.

The second journal to which we have alluded, if its plates of contemporary costume may be relied upon, points to a very curious recent change in the anatomical formation of the female body. The fair originals of the pictures in question could only have attained to the distorted positions in which they are drawn by a series of severe acrobatic efforts, and, even then, could not have maintained such positions except by the aid either of some article of furniture or of a walking stick, although neither one nor the other has been shown by the artists who are responsible for the results. Independently of the difficulty which presents itself to the average male mind, as to how it is possible for any woman removed from idiocy to find pleasure in imitating such hideous caricatures, there is the further question of how, in the present state of knowledge, the trailing skirts can be tolerated as part of a walking dress. They are drawn not only as trailing, but as absolutely lying upon the ground, and it is obviously impossible that the whole of the circumference can be lifted out of contact with the indescribable impurities which it must be the natural province of such skirts to collect and to distribute. It would seem that, as regards women, the increased knowledge of the laws of health which has been gained during the past century has practically been wholly inoperative. Nearly that time has passed away since Abernethy thundered against tight lacing; and the fashions of our own day are just as hideous, just as prejudicial to healthy life and to healthy maternity, as were those of his. Will not some of our great ladies come to the rescue of their long-suffering sex? Will they not endeavour to introduce into this country something of the systematic regard to hygiene which is becoming conspicuous in American education, and which, in proportion as it is real and thorough, must permanently influence for good the physique and the capacity of the race?

